# Retrospective analysis of population-based causes of death and life expectancy in urban Western China from 2003 to 2012

**DOI:** 10.1186/s12889-016-2925-0

**Published:** 2016-03-08

**Authors:** Deng Shi-min, Rong Shun-kang, Yao Yuan-qing, Qian Jun, Huang Jing, Li Nan, Zhou Ren-jiang

**Affiliations:** Second Affiliated Hospital of Chongqing Medical University, Chongqing, China; Department of Chronic and Non-communicable Disease Prevention and Control, Jiulongpo District Center for Disease Control and Prevention, Chongqing, China

**Keywords:** Cause-of-death surveillance, Life expectancy, China, Chongqing, Urban

## Abstract

**Background:**

Hitherto, a population-based analysis of the cause of death in urban areas of Western China has not been undertaken over an extended period. The aims of this study were to calculate the overall and annual cause-specific mortality rates by age and sex in urban areas of Western China from 2003 to 2012 and to evaluate the quality of the data.

**Methods:**

We used Excel software, cause-of-death registrations, and International Classification of Diseases, 10th revision, codes to calculate the overall and yearly cause-specific crude mortality rates by age and sex, the Chinese age-standardized mortality rate, and life expectancies.

**Results:**

In the Jiulongpo District from 2003 to 2012, there was an increase in the number of death case reports in the census-registered population, a decrease in the number of omitted deaths, and rise in the crude mortality rate. Except for 2003, the Chinese age-standardized mortality rate was the lowest in 2012 (330.83/100,000) and highest in 2005 (390.08/100,000). Life expectancy increased from 78.36 years in 2005 to 81.67 years in 2012.

**Conclusions:**

With the development of its social economy, the Chinese government and public attach greater importance to cause-of-death surveillance. The quality of cause-of-death registrations has gradually increased, crude mortality rates have risen, the Chinese age-standardized mortality rate has fallen, and life expectancies have increased.

## Background

Cause of death surveillance is one of the data systems to provide substantial detail about levels and trends in health in China. After establishing and improving cause-of-death registries, we have undertaken the following: analyzed cause-of-death surveillance data over an extended period; described epidemic characteristics, changing trends, and regional disparities in infectious, chronic, and non-communicable diseases and injuries; and quantitatively analyzed factors influencing health. This may provide guidance for policy makers and allow the public to learn about major threats to their health according to their location and adjust their behavior accordingly [1].

Yang Gong-huan and some other scholars use the results of the Global Burden of Diseases, Injuries, and Risk Factors Study 2010 (GBD 2010) to examine in detail the transformation of health in China from 1990 to 2010 and to benchmark health challenges in China to 18 major high-income and rapidly developing countries that are members of the G20 [2]. So a comprehensive and comparable assessment of health challenges and how they change over time is available. The report points out that a national analysis for a country as large and diverse as China could mask substantial variations in key outcomes [2]. So it is necessary to analyze the cause-of-death registration of different provinces. Chongqing is the largest city in Western China, however, as far as the size, social and economic development, and culture are concerned, Chongqing lags behind relative to Beijing and Shanghai. A systematic evaluation of its total population-based cause-of-death statistics and data quality from the start of its cause-of death recording has hitherto not been conducted. The present study statistically analyzed all causes of death in the population and the quality of related data in Jiulongpo from 2003 to 2012. We also offer suggestions for maintaining a population’s health, improving the cause of death registry, and making medical policy decisions.

## Methods

### Object of research

The area covered in the study was a 431.86-square-kilometer urban district in Chongqing. The study included the total census-registered population of Jiulongpo. We analyzed the total number of deaths in the census-registered population; the corresponding index was the mortality rate of that population. The crude mortality rate (CMR) for China and Jiulongpo District have been standardized by the Chinese census-registered population since 2000. Mortality rates of major causes of death and life expectancies at birth in Jiulongpo were studied to analyze the factors influencing the population’s health. Methods of cause of death surveillance and data quality were analyzed to learn about improvements of the cause of death registry. All the population data were provided by the Jiulongpo District Public Security Bureau. Cause-of-death surveillance data were obtained from the Jiulongpo District Center for Disease Control and Prevention (CDC). The authors received permission to use the data to carry out the study upon request to Zhou Ren-jiang who is the director of Jiulongpo CDC.

### Ethical statement

The study is in compliance with the Declaration of Helsinki as described in principle 24—“Privacy and Confidentiality”.

### Methods of information collection

Cause-of-death surveillance has been conducted in Jiulongpo since 1998. The observations for the present study began on January 1, 2003 and ended on December 31, 2012. Death records were obtained from four sources in the district: (i) medical death certificates; (ii) removal from the census-registered population through death—details provided by the Jiulongpo District Public Security Bureau and fatality lists from funeral parlors in Chongqing; (iii) deaths of children under the age of 5 years provided by the Jiulongpo District Center for Maternity and Child Care; and (iv) death reports from rural doctors in their administrative regions. Removals from the census-registered population due to death and fatality lists were acquired by the Jiulongpo District CDC to obtain information including the deceased’s name, sex, age, etc., while information such as cause of death, location of death, methods of diagnosis, etc. were obtained from surveillance workers in hospitals and township health units, household surveys and follow-up communication by telephone which may be conducted when the patient’s relatives are at home or could answer the telephone.

The cause of death was coded using the International Statistical Classification of Diseases, 10th revision (ICD-10), codes. Data from death records were coded and then entered into database by surveillance workers at Jiulongpo CDC after they had collected medical death certificates and death reports for all deaths in the district prior to 2010. They had the access to the Chinese CDC’s Cause of Death Registration System. Deaths were all reported by surveillance workers in all of the hospitals and township health units in Jiulongpo using the Internet after they obtained the total number of deaths in their region beginning in 2010. Annual death reports were statistically analyzed using Cause of Death Registration software developed by Shanghai CDC. All the death records from 2003 to 2012 were compiled and statistically analyzed using Excel 2007 developed by Microsoft Corp.

### Quality control

Quality control of the surveillance was mainly conducted by the Jiulongpo District CDC. After the Jiulongpo District CDC received training in cause-of-death surveillance technology from the Chinese and Chongqing CDCs, it carried out training in the form of meetings and on-site supervision at each hospital and township health unit. The most important task was to review and modify the authenticity, accuracy, and completeness of each death record’s basic information and cause of death as reported by the units. Cause of death surveillance workers in all of the hospitals and township health units were expected to investigate and correct unqualified death reports to maintain the registration’s quality.

The number of all death reports without a clear diagnosis in total and by year from 2003 to 2012 were displayed in Tables 1, 2, 3, 4 and 5. The total number of death reports in Jiulongpo from 2003 to 2012 was 44,894. The composition of the facilities where the deceased’s conditions were diagnosed prior to death were as follows: 17.19 % provincial hospitals; 12.97 % city hospitals; 48.19 % district-level hospitals; 5.72 % township units; 7.32 % rural doctors; and in 8.70 % of cases, there was no visit to a doctor or the diagnostic facility was unknown. The methods of diagnosis were as follows: 0.06 % autopsy; 5.01 % pathology; 0.73 % surgery; 38.60 % clinical and physiochemical tests; and 36.53 % clinical tests. Additionally, 11.29 % of cases were diagnosed after death, and 7.78 % lacked a basic diagnosis. The root causes of death were as follows: 5.50 % due to signs and symptoms not otherwise specified (ICD-10 codes R00–99); 0.03 % not due to an external injury (ICD-10 codes S and T, Y10–34, and Y87.2); 3.02 % due to cardiovascular diseases without diagnostic significance (ICD-10 codes I46._, I47, I49.0, I50, I51.4, I51.5, I51.6, I51.9, and I70.9); 0.11 % due to tumors without a specified location (ICD-10 codes C76, C80, and C97), 0.57 % due to other errors (J96, K72, and N17–19); and 90.77 % of the cases lacked an obvious error.

**Table 1:** Mortality numbers, CMR (/100000), and CASMR (/100000) results for major diseases in Jiulongpo from 2003 to 2012

Diseases	Both Genders			Males			Females		
	Numbers	CMR	CASMR	Numbers	CMR	CASMR	Numbers	CMR	CASMR
Circulatory Diseases	15,028	189.73	108.13	7879	195.98	107.22	7149	183.28	108.96
Malignant Tumors	11,996	151.45	98.17	8138	202.42	127.08	3858	98.91	66.09
Respiratory Diseases	6407	80.89	45.6	4155	103.35	54.9	2252	57.74	33.94
Injuries	3171	40.03	33.75	2144	53.33	45.12	1027	26.33	21.99
Unknown Diagnosis	2471	31.2	17.6	1243	30.92	17.3	1228	31.48	18.05
Endocrine Diseases	1550	19.57	11.82	673	16.74	9.65	877	22.48	14.23
Digestive Diseases	1346	16.99	10.89	882	21.94	13.93	464	11.9	7.67
Diseases of the Nervous System	991	12.51	8.03	554	13.78	8.73	437	11.2	7.23
Urinary Diseases	590	7.45	4.8	330	8.21	5.08	260	6.67	4.45
Infectious Diseases	559	7.06	5.12	386	9.6	6.77	173	4.44	3.34
Perinatal Diseases	227	2.87	4.48	135	3.36	5.45	92	2.36	3.53
Total	44,894	566.78	355.05	26,840	667.61	409.09	18,054	462.86	294.95

**Table 2:** Mortality numbers, CMR (/100000), and CASMR (/100000) results for major diseases in Jiulongpo from 2003 to 2005

Diseases	2003			2004			2005		
	Numbers	CMR	CASMR	Numbers	CMR	CASMR	Numbers	CMR	CASMR
Circulatory Diseases	833	110.76	77.82	941	123.23	83.79	1351	173.79	124.4
Malignant Tumors	818	108.76	81.13	897	117.46	85.1	1043	134.17	101.34
Respiratory Diseases	352	46.8	32.67	471	61.68	41.78	618	79.5	56.83
Unknown Diagnosis	340	45.21	28.85	1211	158.38	107.67	297	38.2	25.54
Injuries	271	36.03	34.27	278	36.4	32.25	254	32.67	29.44
Endocrine Diseases	109	14.49	10.6	99	12.96	9.2	110	14.15	10.39
Digestive Diseases	98	13.03	9.78	63	8.25	6.56	134	17.24	12.94
Diseases of the Nervous System	83	11.04	8.08	60	7.86	5.22	115	14.79	11.55
Urinary Diseases	49	6.52	5.07	36	4.71	3.56	52	6.69	5.3
Infectious Diseases	26	3.46	2.88	31	4.06	3.25	51	6.56	5.34
Perinatal Diseases	15	1.99	3.53	19	2.49	4.1	8	1.03	1.55
Total	3075	408.86	304.12	4141	542.27	387.83	4065	522.9	390.08

**Table 3:** Mortality numbers, CMR (/100000), and CASMR (/100000) results for major diseases in Jiulongpo from 2006 to 2008

Diseases	2006			2007			2008		
	Numbers	CMR	CASMR	Numbers	CMR	CASMR	Numbers	CMR	CASMR
Circulatory Diseases	1397	178.16	109.49	1557	198.34	117.94	1675	210.82	120.45
Malignant Tumors	1098	140.02	95.81	1151	146.62	98.83	1244	156.58	100.53
Respiratory Diseases	691	88.12	54.15	710	90.44	53.48	587	73.88	41.52
Injuries	342	43.61	38.62	354	45.1	39.67	333	41.91	35.38
Unknown Diagnosis	230	29.33	17.38	141	17.96	10.12	62	7.8	4.46
Endocrine Diseases	161	20.53	13.47	159	20.25	12.51	147	18.5	11.57
Digestive Diseases	148	18.87	12.46	159	20.25	13.38	129	16.24	10.24
Diseases of the Nervous System	108	13.77	8.96	106	13.5	9.2	120	15.1	10.15
Urinary Diseases	71	9.05	6.06	95	12.1	8.1	60	7.55	4.99
Infectious Diseases	48	6.12	4.42	45	5.73	3.90	70	8.81	6.9
Perinatal Diseases	14	1.79	3.23	20	2.55	4.47	23	2.89	5.05
Total	4345	554.1	369.97	4532	577.32	377.07	4578	576.21	364.63

**Table 4:** Mortality numbers, CMR (/100000), and CASMR (/100000) results for major diseases in Jiulongpo from 2009 to 2011

Diseases	2009			2010			2011		
	Numbers	CMR	CASMR	Numbers	CMR	CASMR	Numbers	CMR	CASMR
Circulatory Diseases	1594	199.59	106.55	1885	233.5	118.32	1907	231.93	111.01
Malignant Tumors	1224	153.26	94.74	1438	178.13	106.19	1457	177.2	104.52
Respiratory Diseases	700	87.65	47.08	787	97.49	47.93	697	84.77	38.96
Injuries	330	41.32	36.25	346	42.86	32.53	360	43.78	34.64
Endocrine Diseases	148	18.53	10.24	185	22.92	12.23	221	26.88	14.04
Digestive Diseases	134	16.78	9.76	165	20.44	11.79	152	18.49	10.2
Unknown Diagnosis	117	14.65	7.56	23	2.85	1.75	17	2.07	0.86
Diseases of the Nervous System	104	13.02	7.71	108	13.38	8.16	90	10.95	6.44
Infectious Diseases	67	8.39	6.08	56	6.94	4.61	85	10.34	6.74
Urinary Diseases	59	7.39	4.11	71	8.79	5.18	52	6.32	3.38
Perinatal Diseases	32	4.01	6.27	38	4.71	6.58	30	3.65	5.02
Total	4601	576.12	345.82	5120	634.22	358.11	5135	624.53	343.27

**Table 5:** Mortality numbers, CMR (/100000), and CASMR (/100000) data for major diseases in Jiulongpo in 2012

Diseases	Both Genders			Males			Females		
	Numbers	CMR	CASMR	Numbers	CMR	CASMR	Numbers	CMR	CASMR
Circulatory Diseases	1888	225.85	104.12	968	230.3	107.77	920	221.35	106.07
Malignant Tumors	1626	194.51	108.85	1084	257.89	137.56	542	130.41	76.62
Respiratory Diseases	794	94.98	42.60	508	120.86	50.93	286	68.81	31.84
Injuries	303	36.25	26.01	196	46.63	34.64	107	25.74	17.28
Endocrine Diseases	211	25.24	12.57	83	19.75	9.63	128	30.80	16.05
Digestive Diseases	164	19.62	11.40	117	27.84	16.26	47	11.31	6.23
Diseases of the Nervous System	97	11.60	6.09	59	14.04	7.02	38	9.14	5.10
Infectious Diseases	80	9.57	6.78	63	14.99	10.55	17	4.09	2.83
Urinary Diseases	45	5.38	2.81	26	6.19	2.95	19	4.57	2.68
Unknown Diagnosis	33	3.95	2.11	14	3.33	1.60	19	4.57	2.68
Perinatal Diseases	28	3.35	4.37	14	3.33	4.48	14	3.36	4.24
Total	5302	634.24	330.83	3151	749.65	383.1	2151	517.53	273.15

## Results

### Overall mortality

The total census-registered population in Jiulongpo from 2003 to 2012 was 7,920,843 (4,020,336 men, 3,900,507 women). Mortality numbers, CMR, and Chinese age-standardized mortality rate (CASMR) of the total and top 10 diseases resulting in death from 2003 to 2012 in Jiulongpo appear in Table 1. The mortality numbers, CMR, and CASMR results by year from 2003 to 2012 were shown in Tables 2, 3, 4 and 5.

### Overall and annual mortality rates for major diseases for 2003–2012

The mortality numbers, CMR, and CASMR data for major diseases for 2003 to 2012 are displayed in Table 1. The CMR results for circulatory diseases were the highest among all the reported diseases. The proportion of deaths from circulatory diseases among all deaths was 33.47 % from 2003 to 2012. The proportion of deaths from heart disease among all circulatory diseases was 54.25 % (ICD-10 codes I01-25, I27–52). Cerebral diseases accounted for 45.28 % of deaths (ICD-10 codes I60–69). The mortality numbers, CMR, and CASMR data for circulatory diseases by year from 2003 to 2012 were displayed in Tables 2, 3, 4 and 5.

The number of deaths due to malignant tumors (including benign tumors of the central nervous system) was second only to the number of deaths resulting from circulatory diseases. The mortality numbers, CMR, and CASMR results for malignant tumors by year from 2003 to 2012 were shown in Tables 2, 3, 4 and 5. Lung cancer was the most common type of malignant tumor resulting in death in Jiulongpo from 2003 to 2012. The CMR and CASMR for lung cancer were 53.68/100,000 and 33.85/100,000, respectively. Liver cancer, colorectal and anal cancer, gastric cancer, and esophageal cancer were the next-most common types. Detailed information regarding morbidity, mortality, and survival rates of malignant tumors in Jiulongpo have been presented in previous reports [3–5].

Injuries and toxicosis were the fourth-most common causes of death. Deaths due to accidents caused by motor and non-motor vehicles were the most frequent cause of death by injuries and toxicosis. In all, 1115 deaths due to traffic accidents were reported from 2003 to 2012, giving a CMR for traffic accidents of 14.08/100,000 and CASMR of 11.91/100,000.

Infectious diseases ranked ninth among all causes of death in Jiulongpo. Tuberculosis (TB) was the most common infectious disease causing death. The total number of deaths caused by TB was 172 from 2003 to 2012, with a CMR of 2.17/100,000 and CASMR of 1.50/100,000. The CMR of TB was reported to be 1.60/100,000 in Jiulongpo in 2003, 1.96/100,000 in 2004, and 1.91/100,000 in 2012. Chronic hepatitis B was the second-most common infectious disease causing death, with 142 total deaths—a CMR of 1.79/100,000 and CASMR of 1.25/100,000 from 2003 to 2012. The CMR of chronic hepatitis B was reported to be 2.25/100,000 in 2009 and 1.91/100,000 in 2012. Additionally, 55 deaths due to AIDS were reported from 2003 to 2012, giving a CMR of 0.69/100,000 and CASMR of 0.57/100,000. The CMR of AIDS was reported to be 0 in 2003, 0.13/100,000 in 2004, and 2.27/100,000 in 2012.

The overall CMR and CASMR for children under the age of 5 years were 9.47/100,000 and 14.80/100,000, respectively. The CMR for children under the age of 5 was observed to be 5.45/100,000 in 2003, 7.99/100,000 in 2004, and 6.34/100,000 in 2012.

The overall CMR and CASMR values for maternal deaths (maternal deaths in this study include direct and indirect death during pregnancy and within 6 weeks of delivery) were 0.06/100,000 and 0.07/100,000, respectively. Except for one case of maternal death reported in each of 2004, 2006, 2007, 2009, and 2010, there were no reported cases of maternal death in the other years.

### CASMR and life expectancy for 2003–2012 in Jiulongpo

The census-registered population in Jiulongpo was 752,084 in 2003, 763,635 in 2004, 777,382 in 2005, 784,146 in 2006, 785,007 in 2007, 794,507 in 2008, 798,618 in 2009, 807,286 in 2010, 822,223 in 2011, and 835,955 in 2012. The life expectancy values in total and by year for the census-registered population in Jiulongpo appear in Table 6. Except for 2003, the CASMR was lowest in 2012 and highest in 2005. Similarly, except for 2003, life expectancy was highest in 2012 (81.67 years) and lowest in 2005 (78.36 years).

**Table 6:** Life expectancy from 2003 to 2012 in Jiulongpo

Gender	2003	2004	2005	2006	2007	2008	2009	2010	2011	2012	2003 ~ 2012
Male	80.63	76.99	75.27	77.28	77.07	77.18	77.36	76.93	78.22	79.01	77.62
Female	86.68	82.43	81.86	82.56	83.11	82.67	84.57	83.88	84.39	84.64	83.64
Both	83.51	79.53	78.36	79.77	79.90	79.77	80.68	80.17	81.13	81.67	80.45

### CMR results for different age-groups

**Fig. 1:**
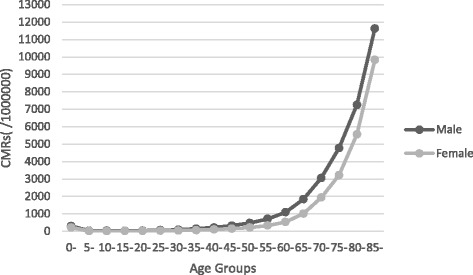
Crude mortality rates of different age groups of males and females in Jiulongpo from 2003 to 2012

Figure 1 presents the CMR results for different age-groups for both males and females. The CMR increased with age. The CMR results for individuals aged 65–70 years show a rise for both males and females, though the CMR for males was higher. The CMRs for both genders reached a maximum at age 85 years and above. Those values were 11,629.03/100,000 and 9832.86/100,000 for males and females, respectively.

## Discussion

Jiulongpo is located southwest of Chongqing. At the end of 2012, the census-registered population was 835,955, made up of 650,855 urban and 185,100 rural residents. Table 5 lists the overall CMR, CASMR, and top 10 diseases of Jiulongpo in 2012. The CMR for permanent residents at all of the monitoring sites in Chongqing was 635.24/100,000. The top 10 diseases and their CMRs were as follows: circulatory diseases (250.20/100,000), tumors (165.83/100,000), respiratory diseases (125.87/100,000), injuries and toxicosis (55.67/100,000), digestive diseases (17.89/100,000), endocrine diseases (13.27/100,000), infectious diseases (10.50/100,000), urinary diseases (6.27/100,000), other diseases (5.57/100,000), and diseases of the nervous system (3.98/100,000). The CMR and CASMR for all monitoring sites in China in 2012 were 592.52/100,000 and 419.34/100,000, respectively. The top 10 diseases and their CMRs were as follows: circulatory diseases (254.94/100,000); malignant tumors (141.23/100,000); respiratory diseases (79.03/100,000); injuries (50.16/100,000); digestive diseases (13.68/100,000); endocrine and metabolic diseases (12.02/100,000); urinary diseases (6.61/100,000); infectious diseases (6.59/100,000); diseases of the nervous system (5.76/100,000); and perinatal diseases (3.12/100,0000) [6]. These findings demonstrate that Jiulongpo was one of the more urban districts of Chongqing and that Jiulongpo’s death rate reflected the health and diseases patterns seen in large cities.

The Global Burden of Disease Study 2013 (GBD 2013) [7] found that the number of deaths for most of the leading non-communicable diseases increased by 42 % from 1990 to 2013; however, age-standardized mortality rates fell. Age-standardized death rates from non-communicable diseases fell by 18.6 %, and by 22 % for cardiovascular and circulatory diseases. The aging and growth of populations has led to an increase in the total number of cardiovascular deaths, which in 2013 accounted for almost a third of all deaths globally [7]. From 2003 to 2012 in Jiulongpo, the CMR of circulatory diseases increased and the CASMR decreased slightly. The reason for this could also be the aging and growth of the population. More deaths were caused by heart than by cerebral diseases. This finding is consistent with the report of Li -Qi [8]. Almost no difference was observed between males and females in the CMR and the CASMR, which indicates that circulatory diseases are equally damaging to men and women.

With malignant tumors, both the CMR and CASMR increased from 2003 to 2012 in Jiulongpo. That finding is somewhat different from the GBD 2013 results, which identified a fall in age-standardized cancer death rates [7]. In Jiulongpo, both the CMR and CASMR for males were almost twice those of females. Among males, the CMR for malignant tumors was even higher than that for circulatory diseases. Malignant tumors were notably more harmful for men than women. The disparity between the CMRs for males and females was not only due to genetics and lifestyle, but also because the incidence of highly malignant lung and liver cancers with low survival rates was higher in males than females [4, 5].

The present study found that traffic accidents of all kinds caused the greatest number of deaths among all analyzed external causes. One way to reduce the number of deaths caused by traffic accidents would be to enforce strict adherence to traffic rules.

We found a decrease in the death rates of TB and hepatitis B. However, the deaths caused by AIDS did not follow that trend: the CMR for AIDS increased in Jiulongpo from 2003 to 2012, and the actual mortality rate may have been even higher. Since Human Immunodeficiency Virus (HIV) testing, Syphilis testing, hepatitis B testing and other virus testing is not necessary for the patients at a hospital if they are not treated by operation, the doctors won’t know whether the patients are infected by HIV, when inpatients with AIDS and their relatives conceal their condition because of social prejudice, making the reported death rate lower than the actual one. Methods for increasing knowledge regarding the prevention and control of AIDS, ensuring accuracy and integrity of AIDS incidence and death records, and protecting the privacy of patients are very important to future public health work, as social and other problems caused by AIDS will not be fewer than those caused by chronic non-communicable diseases such as circulatory diseases and tumors. Our findings related to the epidemic characteristics of TB and AIDS deaths are consistent with those of previous reports [2, 9].

Child mortality is decreasing worldwide and has been decreasing in many countries for many decades [10–19]. China is a case in point. The CMR of individuals under 5 years of age in Jiulongpo was lower than the reported national mortality rate [19].

Reduction of maternal mortality has long been a global health priority and is a target in the United Nations Millennium Development Goals framework [20]. Some progress toward this target has been reported, especially in the past decade [21–24]. The findings of GBD 2013 indicate that the rate of change in maternal mortality in some developing countries, e.g., China, has exceeded 8 % over the past decade [25]. The present study demonstrated that the maternal death rate in Jiulongpo was lower than the reported national mortality [25].

Life expectancy can objectively reflect economic development, medical care, and the health of a local population because the decline in mortality is greater in richer or less-deprived areas [26]. In Khan’s and Asaduzzaman’s study, the obtained results at the national level shows the remarkable difference in the Literate Life Expectancy between urban and rural people (men and women) [27]. The GBD 2013 found that life expectancy has improved faster than the global aggregate trend in China and Chile in the past 23 years [7]. The average life expectancies for residents of Shanghai, Beijing, and Tianjin in 2011 were 82.13, 80.80, and 80.65, respectively. The life expectancy for permanent residents in Chongqing was 78.54 years in 2012 (Data of life expectancies is from《Report of cause-of-death registration of Chongqing in 2012》). The overall and annual life expectancies in Jiulongpo from 2003 to 2012 appear in Table 6. The life expectancy in Jiulongpo was 80.17 in 2010 and 81.13 in 2011, that is higher than the average national life expectancy [2], and is similar to the average life expectancies of residents in Shanghai, Beijing and Tianjin. That figure is also close to the average nationwide life expectancies of high-income countries, such as Finland and Germany [28], though it is lower than those of France, Canada, and Japan [16].

From 2003 to 2012, the number of death reports rose from 3075 to 5302, the CMR rose from 408.86/100,000 to 634.24/100,000, and the CASMR fluctuated between 304.12/100,000 and 390.08/100,000. The increase in the CMR reflects the decrease in missing death reports, which is supported by the absolute increase in the number of death reports. Four sources of death reports were described in materials and methods section, and the form of information collection reflected the method of recording causes of death in most developing regions in China. Even medical certification of causes of death has limitations, which is shown by the need for garbage code redistribution [29–31]. However, medical certification is more accurate than obtaining causes of death out of hospital. When cause of death surveillance began, the proportion all death reports that came from medical death certificates from hospitals was only 40.2 %. The other 60 % of cases involved deaths that occurred out of hospital. There were more omissions and inaccuracies at the early stage of the death surveillance work. As displayed in Table 2, the CMR in 2003 was only 408.86/100,000. The life expectancy was as high as 83.51 in 2003, mainly because there were so many death reports omitted. Although the CMR rose to 542.27/1,000,000 in 2004, the number of deaths without a clear diagnosis was 1211. Jiulongpo CDC obtained death reports from Jiulongpo Security Bureau to ensure the integrity of death reports, but information as to the cause of death in those cases was unclear. The CMR increased more in 2004 than it did in 2003, but the proportion of unknown diagnoses was as high as 29.24 %. The reason for this was unrecorded deaths. Since 2005, Jiulongpo CDC has been in regular communication with Jiulongpo Security Bureau. Workers from Jiulongpo CDC send a list of deaths based on removals from the census to hospitals and township health units in the Jiulongpo District. Workers in the units collect information such as the cause of death, diagnostic findings, etc. using household surveys. As a result, the proportion of death reports without clear diagnoses dropped to 7.31 % after 2005. In 2010, with the help of policy recommendations from Chongqing Health Bureau, Chongqing Municipal Government issued a new regulation calling for multiple departments in the city to work together to regulate cause of death reports. The policy required that family members of those who died at home obtain a medical death certificate from a doctor at a hospital. At the same time, Jiulongpo CDC began contacting the Department of Funeral and Civil Affairs to further increase the information collected about deaths. As a result, the size and quality of the cause of death registry has increased significantly, and the CMR has risen to 634.22/100,000. Since 2010, the proportion of deaths reported from medical death certificates has been 84.85 % of all death case reports. The remaining death reports were derived from information provided by Jiulongpo Security Bureau, Funeral Department and rural doctors. There has been a qualitative leap between the beginning stages and the present in terms of the amount and quality of death reports. Improvements in data quality can be attributed to the method of information collection that Jiulongpo CDC developed as well as the increased importance the government and society attached to cause of death surveillance. Additionally, it is a basic reflection of social development.

In the present study, we obtained information only about cause-specific mortality rates by age and gender. Further research is required about what might appropriately be regarded as premature mortality, years of life lost due to premature mortality, and the relationship between mortality and population change.

## Conclusions

With social development, there has been a gradual increase in the quality of cause-of-death registration in Jiulongpo as well as a rise in the CMR of all causes of death, decrease in the CASMR, and increase in life expectancy. This study found that among all the circulatory diseases examined, more deaths were caused by heart conditions than by cerebral diseases in Jiulongpo. We observed almost no differences between males and females in the CMR and CASMR for circulatory diseases; whereas among males, both the CMR and CASMR for malignant tumors were almost twice those of females. From 2003 to 2012, there was an increase in the CMR for AIDS. The life expectancy in Jiulongpo was 81.67 in 2012.

## Abbreviations

GBD 2010: Global Burden of Diseases, Injuries, and Risk Factors Study 2010
G20: the Group of twenty countries
CDC: Center for Disease Control and Prevention
CMR: the crude mortality rate
ICD-10: International Statistical Classification of Diseases, 10th revision
CASMR: Chinese age-standardized mortality rate
TB: Tuberculosis
AIDS: Acquired Immune Deficiency Syndrome
GBD 2013: The Global Burden of Disease Study 2013
HIV: Human Immunodeficiency Virus
